# Micro-geographic risk factors for malarial infection

**DOI:** 10.1186/1475-2875-8-27

**Published:** 2009-02-13

**Authors:** Ward P Myers, Andrea P Myers, Janet Cox-Singh, Hui C Lau, Benny Mokuai, Richard Malley

**Affiliations:** 1 Infectious Disease, Children's Hospital Boston, Boston, USA; 2 School of Medicine, Stanford University, Stanford, USA; 3 Malaria Research Centre, University of Malaysia Sarawak, Kuching, Malaysia; 4 Ambunti Health Centre, Ambunti, Papua New Guinea

## Abstract

**Background:**

Knowledge of geography is integral to the study of insect-borne infectious disease such as malaria. This study was designed to evaluate whether geographic parameters are associated with malarial infection in the East Sepik province of Papua New Guinea (PNG), a remote area where malaria is a major cause of morbidity and mortality.

**Methods:**

A global positioning system (GPS) unit was used at each village to collect elevation, latitude and longitude data. Concurrently, a sketch map of each village was generated and the villages were sub-divided into regions of roughly equal populations. Blood samples were taken from subjects in each region using filter paper collection. The samples were later processed using nested PCR for qualitative determination of malarial infection. The area was mapped using the GPS-information and overlaid with prevalence data. Data tables were examined using traditional chi square statistical techniques. A logistic regression analysis was then used to determine the significance of geographic risk factors including, elevation, distance from administrative centre and village of residence.

**Results:**

Three hundred and thirty-two samples were included (24% of the total estimated population). Ninety-six were positive, yielding a prevalence of 29%. Chi square testing within each village found a non-random distribution of cases across sub-regions (p < 0.05). Multivariate logistic regression techniques suggested malarial infection changed with elevation (OR = 0.64 per 10 m, p < 0.05) and distance from administrative centre (OR = 1.3 per 100 m, p < 0.05).

**Conclusion:**

These results suggest that malarial infection is significantly and independently associated with lower elevation and greater distance from administrative centre in a rural area in PNG. This type of analysis can provide information that may be used to target specific areas in developing countries for malaria prevention and treatment.

## Background

Knowledge of geography is integral to the study of disease within populations. This is particularly true for insect-borne diseases, such as malaria, because transmission depends on an interaction with a vector that has a limited geographical range. Macrogeographic trends in disease prevalence have been recognized; as an extreme example, malaria in man is endemic in sub-Saharan Africa, but is not transmitted in Greenland. Microgeographic variability in disease prevalence is often overlooked but is still an important determinant of disease. For example, in villages in malaria endemic regions, some neighbourhoods have a high malaria burden while others seem to be disease-free[1,2]. However, as malaria endemicity is particularly common in remote, un-mapped, impoverished areas, which lack street addresses and even basic census information, the scientific analysis of these microgeographic trends has been historically limited. Newer technologies such as detailed satellite imagery, hand-held geographic position sensing devices, and computerized geographic information systems, are now making this analysis possible.

The importance of microgeographic risk factors is underscored when one considers effective strategies for reducing malaria in resource-poor settings. Asymptomatic adult carriers of malarial parasites act as reservoirs within communities, placing children and others who are more susceptible, at risk for infection and more severe disease. As these individuals may not feel sick, they typically do not present to health care settings where they can be treated. Given that the total health care expenditure in Papua New Guinea is $30 US per person, per year (based on average exchange rates)[3], individualized screening is not possible. However, if microgeographic risk factors for malarial infection can be specifically identified, the limited health care resources can be focused on properly delineated areas, reducing the cost of control measures while reducing thethe ovel burden of malaria disease.

## Methods

### Study site

Geographically, the East Sepik Province of Papua New Guinea is an immense river basin comprising jungles, swamps, and grasslands. According to the Köppen climate classification system, the region is classified as tropical rain forest (Af) and annually has up to seven meters of rainfall[4,4]. The year is divided into a "dry" season (May through September) in which regular rain occurs without flooding and a "wet" season (October through April) in which rain causes the river to overflow its banks and flood most villages. Samples were collected for this study in July 2004.

In the upper Sepik, most communities are scattered along the banks of the Sepik River or one of its many tributaries. The economy is primarily based on subsistence farming with some hunting and gathering. It is quite rural with transport occurring on foot or by boat. Data used in this study were collected from two representative villages, Oum and Ambunti, in the Upper Sepik region of the East Sepik Province. Ambunti is a larger village, situated on the main Sepik River. It has a large airstrip and serves as a regional "capital" and economic hub of the region. Oum is a smaller village, situated on an ox-bow off the main Sepik River. It is a several day hike or a six hour motorized canoe ride between the two villages.

Malaria in this region is considered hyper-endemic[5]. Studies elsewhere in Papua New Guinea have documented infections with all of the four Plasmodium species that commonly infect humans (*Plasmodium falciparum, Plasmodium vivax, Plasmodium malariae *and *Plasmodium ovale*). Published data from less remote villages in the East Sepik Province have shown malarial infection rates near 30%[6]. Previous unpublished data from this region found a rate of malarial infection closer to 50%.

### Data collection

Blood samples were collected from two different village regions. The first set of samples was collected from the less accessible village, Oum. An initial sketch map was drawn of the village by hand. Geographic features were used to divide the village into 10 zones. Each zone had an estimated population of 50 individuals. Individuals tended to live in multi-family dwellings with fifteen people per dwelling. Within each zone, all houses were numbered and one was selected by a random number-generating device. Individuals living in that house were given the first opportunity to participate in the study. Sampling then progressed to the next nearest house until 20 subjects were given the opportunity to participate or the number of potential subjects was exhausted. Blood was collected from 142 subjects, or 28% of the estimated population of Oum.

In Ambunti, the more accessible village, a similar sampling strategy was employed. However, as the population density of Ambunti was higher, similar mapping techniques resulted in fewer, but more populous zones. Four zones had an estimated population of 200 individuals and the fifth zone had an estimated population of 100 individuals. In each zone 50 (or 25 in the smaller zone) individuals were given the opportunity to participate. Blood was collected from 221 subjects or 25% of the estimated population of Ambunti.

Samples were collected in the early evening to maximise the number of people at home. From each individual, approximately 200 microliters of blood was collected onto Whatman filter paper. The samples were allowed to air dry and then stored on plastic bags. All samples used in this study were collected by the local health volunteers. Oral informed consent was taken prior to sample collection. Subjects were not compensated for sample collection, and to assure confidentiality, no identifying information other than location was recorded for each blood sample. This study was approved by the Stanford University Institutional Review Board and the Papua New Guinea Medical Research Advisory Council.

Geographic data was collected using a Garmin Etrex Venture handheld GPS device (Garmin International, Olathe, KS). Within each village elevation, latitude, and longitude were collected for points of interest, foot-paths, rivers, and other natural boundaries. Data for linear and polygonal features such as rivers and foot-paths were recorded using points collected approximately every 10 meters. This geographic data was then imported into ARC Info version 9.2 (Redlands, CA). Detailed maps of the two villages were then created from the GPS data. Information from the initial hand-sketched maps was then incorporated into the detailed GIS map, allowing digital mapping of the zones used for sample collection. An approximate mean elevation was calculated for each zone by averaging the elevation of all recorded points on foot-paths within that zone (range 67 to 132 meters above sea level). For each zone, a centre point was calculated by the ARC Info software. This centre point was used to calculate distances between each zone and the village administrative centre (range 19 to 791 meters) and between each zone and the river (range 32 to 855 meters). The village administrative centre was defined as the district where the community radio and town leader's offices were located. Each zone's elevations and distance measurements were used as proxy measurements for samples collected within that zone.

### Molecular and statistical analysis

The DNA was extracted from all of the blood samples using a modified protocol from Instagene (Bio-Rad Laboratories, Hercules, CA). Briefly, 2 holes (the equivalent of 20 microliters of blood) were "punched-out" of each dried blood spot. The samples were then re-hydrated in 200 microliters of Instagene, incubated at 55C for 1 hour, boiled for 8 minutes and centrifuged for 3 minutes. The supernatant (the extracted DNA) was removed and stored at -20°C. The extracted sample served as the template for PCR analysis. PCR products were analysed by standard agarose gel electrophoresis, ethidium bromide staining, and visualization by UV excitation Sample collection and processing protocols as well as sequences of oligonucleotide primers have been previously published[7,8]. For every set of 15 samples extracted, both positive and negative control samples were included. Positive controls had known low or high parasite concentrations to control for sensitivity and specificity. All samples were analysed using Plasmodium genus-specific nested PCR to detect all cause malaria positive samples including *P. falciparum*, *P. vivax*, *P. malariae *and *P. ovale*. All positive controls were positive, and all negative controls were negative. Thirty-one samples produced bands that were fainter than the low level positive control. As the low positive band was set near the standard detection threshold for this assay, these samples were excluded.

Because of geographic and population differences, the two villages were initially analysed separately. A Chi square test was used to determine if cases were non-randomly distributed among the zones in each village. Given that some sub-regions had fewer than five positive cases, Fisher's exact test was used to confirm the Chi square results. Univariate odds ratios for malarial infection were calculated by logistic regression for elevation, distance from the river bank, and distance from the administrative centre. Multivariate logistic regression was then performed to evaluate independent predictors of the individual risk of malarial infection. Malarial infection was also analysed by zone using a Poisson based regression model. This model showed similar trends to the logistic model, but with a decrease in significance that corresponded to the larger unit of analysis. Given that the analyses of both villages exhibited similar trends for each of the three variables, the two data sets were combined into a single analysis for increased statistical power. All analyses were conducted in Stata 7.0 (College Station, TX).

## Results

**Figure 1:**
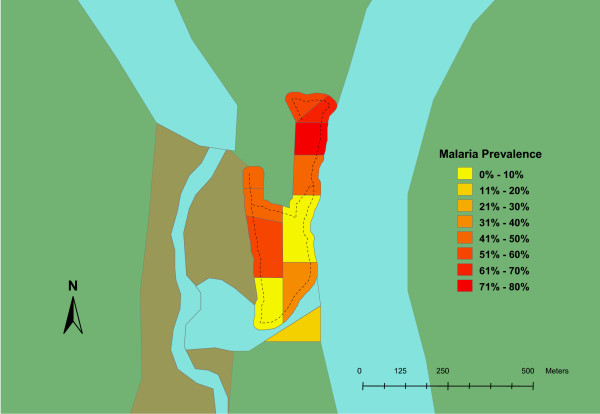
**Prevalence map for malarial infection in Oum village**. Zones within the village are color coded from low (yellow) to high (red) prevalence. Other geographic features: river, dry riverbed, and jungle are colored blue, brown and green respectively. Main footpaths are represented by dotted lines.

**Figure 2:**
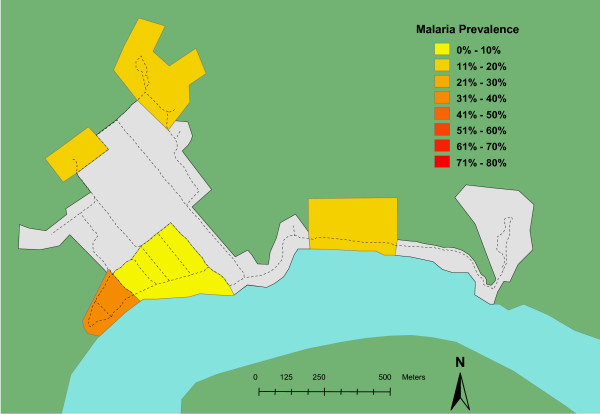
**Prevalence map for malarial infection in Ambunti village**. Zones within the village are color coded from low (yellow) to high (red) prevalence. Other geographic features: river and jungle are colored blue and green respectively. Main footpaths are represented by dotted lines. Grey areas represent zones within the village with very low residential population densities (such as airstrip and religious/commercial districts.).

Blood samples from 332 individuals were included in this study. This represented twenty-two percent of the total estimated population for these two villages. In Oum, 63 of the 128 (49%) samples were positive. In Ambunti, 33 of the 204 (16%) samples were positive. Zones within each village also displayed significant variability (Table 1). The spatial distribution of malaria prevalence in the two villages, Oum and Ambunti, is presented in Figures 1 and 2 respectively. Chi square testing, stratified by village, found that cases of malarial infection were not randomly distributed among the zones (p < 0.05 for each village).

**Table 1:** Geographic data and percent infected by zone

**Zone**	**Village**	Elevation*	Distance from village center*	Distance from river bank*	**Percent infected**
1	Oum	100	242	40	0
2	Oum	100	106	140	57
3	Oum	101	113	153	47
4	Oum	116	182	96	46
5	Oum	102	174	39	40
6	Oum	105	19	34	0
7	Oum	102	149	34	46
8	Oum	105	256	50	71
9	Oum	91	340	32	69
10	Oum	100	356	90	58
11	Ambunti	67	399	83	31
12	Ambunti	127	687	603	12
13	Ambunti	75	198	145	4
14	Ambunti	132	791	855	12
15	Ambunti	75	582	101	15

*elevations and distances are in meters

### Univariate analysis

In Oum, the univariate odds of malarial infection escalated significantly (p < 0.05) with increasing distance from the administrative centre of the (Table 2). Other notable, but not significant, trends in the village of Ambunti included less malaria as elevation increased and one moved away from the river.

**Table 2:** Univariate associations with malarial infection

	**Oum**		**Ambunti**	
	**Odds Ratio**	**95% CI**	**Odds Ratio**	**95% CI**
**Elevation (per 10 m)**	0.72	[0.39, 1.31]	0.88	[0.77, 1.02]
**Distance from village centre (per 100 m)**	1.58*	[1.11, 2.26]	0.92	[0.76, 1.12]
**Distance from river bank (per 100 m)**	1.32	[0.6, 2.89]	0.91	[0.8, 1.03]

* significant at the p < 0.05 level

### Multivariate analysis

After adjusting for differences in the other variables by multivariate analysis, the two villages displayed similar trends. Odds of malarial infection increased with decreasing elevation, increasing distance from the village administrative centre, and increasing distance from the river (Table 3). Given the parallel findings in both villages the data were collapsed into a single analysis for increased power. In the pooled analysis, both lower elevation and further distance from the village centre were significantly associated with increased malaria prevalence, while no significant association was found with distance from the river.

**Table 3:** Multivariate associations with malarial infection

	**Oum**		**Ambunti**		**Pooled**^‡^	
	**Odds Ratio**	**95% CI**	**Odds Ratio**	**95% CI**	**Odds Ratio**	**95% CI**
**Elevation (per 10 m)**	0.92	[0.49, 1.73]	0.6	[0.27, 1.31]	0.64*	[0.41, 0.99]
**Distance from village centre (per 100 m)**	1.71*	[1.14, 2.57]	1.19	[0.84, 1.69]	1.3*	[1.03, 1.66]
**Distance from river bank (per 100 m)**	1.93	[0.82, 4.53]	1.31	[0.69, 2.49]	1.2	[0.82, 1.76]

* significant at the p < 0.05 level

^‡ ^Data from both villages combined. Village also was included as cofactor in multivariate analysis given its strong association with malarial infection.

## Discussion

This study found that malaria cases were not evenly distributed throughout the research area. One village, Oum, had a higher prevalence of malarial infection, and within each village cases were more common in particular zones. In particular, areas at lower elevations and those further from the village centre had more cases of infection. These geographic trends present in the univariate analysis were confirmed and noted to be significant in the multivariate models.

Differences in disease prevalence for vector-based infections such as malaria are generally thought to be consequent to one of two causes: differences in the vector population or the host population. These same categories can be applied to the microgeographic associations detected in this study.

There are several vector-related dynamics that could contribute to the associations observed in this study. The *Anopheles punctulatus *mosquito, the primary vector of malaria in the Sepik Region of Papua New Guinea [9], has been shown to have an uneven spatial distribution within villages[10]. This would permit differences in the vector population to contribute to the micro-geographic differences found in malarial infection. Elevation is known to be associated with differences in mosquito population and malaria cases on a larger scale[11,12]. Alternatively, elevation in this study may be a proxy for water accumulation. Zones with higher mean elevations tended to be hillier with fewer flat areas for water to pool. Similar geographic research in Kenya[13] found that both elevation and wetness indices were independently associated with microscopically-confirmed malaria cases. The increasing rate of malarial infection with greater distances from the village centre could also be secondary to proximity to mosquito breeding sites. Residences on the village outskirts would potentially be located closer to swamps and agricultural fields, which might result in a greater local mosquito density.

Alternatively these microgeographic trends could be related to differences in the human population. Socio-economic status can be protective against malaria if it provides better access to anti-malarial medicines or bed nets [Ward I agree with you but I think this statement would need to be supported by a reference if possible]. In this area, it is possible that the more established families with more resources live near the town centre and/or on higher ground. Similar patterns (residence elevation correlated with affluence) have been observed elsewhere, most notably in pre-hurricane Katrina New Orleans. Previous malaria work has found that proximity to village centre and higher socio-economic status were associated with less malaria[14]. These trends could also be explained non-economically by the proximity of health clinics and markets that sell bed nets and anti-malarial medications to the village centre; the relatively mild symptoms of malaria in recurrently infected adults may not be sufficient incentive to prompt a trip to the health center.

Previous large studies of geographic associations with malaria prevalence have found a positive association with proximity to rivers and increased mosquito density and malaria [15-17]. Distance from the river was not noted to be significant in the present study. This could be due to a variety of factors. Many of the previously mentioned studies looked for geographic associations on a much larger scale. This effect might be less robust when examined over a few hundred meters. Additionally, the local environment in the Sepik river basin includes many stagnant water swamps and other shallow pools and streams. In this area of Papua New Guinea, these other bodies of water may be more important for mosquito breeding than the relatively faster moving Sepik River.

Although each village exhibited similar trends in microgeographic risk factors, the overall levels of malarial infection differed significantly between the two villages. The proportion of infected subjects was more than doubled in Oum as compared to Ambunti. This finding is consistent with the authors' unpublished survey data from the region. This difference is likely secondary to Ambunti's far more accessible location (resulting in greater economic opportunity and better access to antimalarials and bednets), but differences in vector populations cannot be discounted.

This work is not without its weaknesses. While the overall participation rate was quite high, a systematic bias in sample collection is always a possibility in this type of survey. Blood samples were collected in the early evening (when most family members would be at home) to minimize sampling bias. To the extent that transmission occurred outside the subject's zone (e.g. in the agricultural fields away from the village), some misclassification may have occurred, but as a majority of *Anopheles punctulatus *bites occur between 2 AM and 6 AM this type of misclassification was minimized [9]. As this is primarily an agricultural area, hunting outside the village at night tends to be uncommon and is largely limited to adult males. To the extent that the residences of these males are non-randomly distributed throughout the villages, this might serve as an additional confounder.

Additionally, some precision in data was lost by using the geographic centre of the zone in which a subject resided as a proxy for subject's geographic data. The ideal study of microgeographic risk would use the exact coordinates of the location where the malarial infection occurred at the unit of geoanalysis. As this degree of precision is not yet practical, most present studies aggregate subjects by individual residence, village, or district, and use this information as a proxy for the subjects geographic data[14,18,19]. This study aggregated subjects into zones that consisted of a small number of communal housing structures. This unit of analysis was chosen because it struck an appropriate balance between the desired geographic precision and the realities of data collection in an unmapped resource poor setting.

Finally, it remains to be seen if the microgeographic associations found in this study would have use in other malarious areas. This region of Papua New Guinea has many unique environmental, economic, and geographic aspects, and one might expect to find different trends in different regions. More importantly, however, the existence of such microgeographic associations underscores the importance of tailoring a malaria control programme to meet local needs.

## Conclusion

The risk of malarial infection can vary over short distances and small changes in elevation. In this region of Papua New Guinea, increasing distance from the village centre and decreasing elevation were positively associated with this risk. As malaria is largely a disease of the rural poor, knowledge of these microgeographic trends is critical to efficiently apportion limited resources in the struggle against malaria.

## Competing interests

The authors declare that they have no competing interests.

## Authors' contributions

WPM designed the study, conducted the fieldwork, analysed the data, and drafted the manuscript. APM was involved in study design, molecular analysis and manuscript preparation. JCS advised on methodology and manuscript preparation. HCL assisted with blood sample processing and PCR analysis. BM assisted with field logistics and sample collection. RM assisted with study design and manuscript preparation. All authors read and approved the final manuscript.
